# Metachronous small bowel adenocarcinoma after rectal adenocarcinoma: A case report

**DOI:** 10.1097/MD.0000000000027448

**Published:** 2021-10-08

**Authors:** Ya’nan Zhen, Jianqi Li, Ruogu Wang, Shoutang Lu, Yongshun Zhou, Ruixue Xiao

**Affiliations:** a Department of Gastrointestinal Surgery, The Third Affiliated Hospital of Shandong First Medical University, Affiliated Hospital of Shandong Academy of Medical Sciences, Jinan, Shandong, P.R. China; b Gastroenterology Institute and Clinical Center of Shandong First Medical University, Jinan, Shandong, P.R. China; c Department of Pathology, The Third Affiliated Hospital of Shandong First Medical University, Affiliated Hospital of Shandong Academy of Medical Sciences, Jinan, Shandong, P.R. China.

**Keywords:** case report, intestinal obstruction, metachronous, small bowel adenocarcinoma, small bowel cancer

## Abstract

**Rationale:**

Small bowel adenocarcinoma (SBA), an uncommon gastrointestinal malignant tumor, is difficult to diagnose at an early stage because of its non-specific disease presentation. Metachronous SBA is a special type of SBA that is rarely reported. We herein report a case of metachronous primary SBA following resection of rectal adenocarcinoma.

**Patient concerns:**

A 65-year-old man presented to our hospital after having experienced recurrent bowel obstruction for 6 months. He had undergone a Dixon operation 30 months previously followed by adjuvant chemotherapy with capecitabine plus oxaliplatin.

**Diagnosis:**

Abdominal computed tomography showed thickened bowel walls in the right lower abdomen, and the patient was initially misdiagnosed with intestinal adhesion. After the operation, he was diagnosed with primary SBA (T3N0M0, stage IIA).

**Interventions:**

Treatment with a transnasal ileus tube was ineffective. Therefore, we performed small intestinal segmental resection and side-to-side anastomosis through open surgery.

**Outcomes:**

The patient completed all postoperative adjuvant chemotherapy, and posttreatment surveillance revealed no further abnormalities.

**Lessons:**

This case suggests that patients with colorectal adenocarcinoma may have an increased risk of metachronous SBA. Corresponding symptoms in high-risk patients should raise clinicians’ suspicion for SBA, and further detailed examinations are imperative. Early screening for SBA may help to improve the patients’ prognosis.

## 1 Introduction

Small bowel cancer is a rare gastrointestinal malignant tumor accounting for only 0.6% of all cancer diagnoses and less than 5% of all gastrointestinal cancer diagnoses in the United States.^[1]^ The early symptoms of small bowel adenocarcinoma (SBA) are often latent and non-specific abdominal symptoms, including abdominal pain, nausea, vomiting, gastrointestinal bleeding, and intestinal obstruction. Most patients with SBA are diagnosed at an advanced stage and thus have a poor prognosis.^[2]^ Patients with colorectal cancer (CRC) have an increased risk of metachronous SBA.^[3]^ We herein report a case of metachronous SBA that was diagnosed 30 months after rectal adenocarcinoma.

## 2 Case presentation

A 65-year-old man had a surgical history of a Dixon operation for treatment of rectal cancer in February 2018. Histopathological examination revealed moderately differentiated rectal adenocarcinoma with involvement of the deep muscle layer. No metastatic carcinoma was observed in the peripheral lymph nodes (0/35), mesenteric lymph nodes (0/20), intestinal wall lymph nodes (0/13), or anterior abdominal aortic lymph nodes (0/2); however, a cancerous nodule was found in the subserous adipose tissue. The pathological stage was pT2N1M0, IIIA. Six cycles of chemotherapy with capecitabine plus oxaliplatin were performed after the operation.

**Figure 1:**
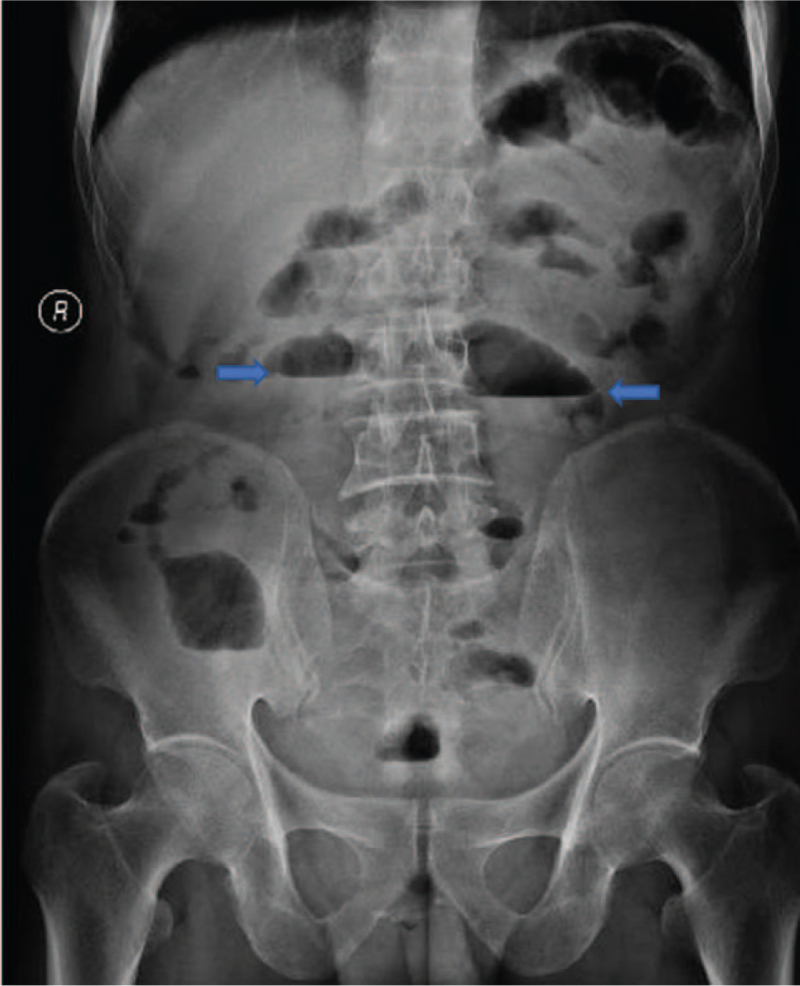
Abdominal X-ray showed an intestinal obstruction (arrows indicate the multiple gas–liquid planes).

**Figure 2:**
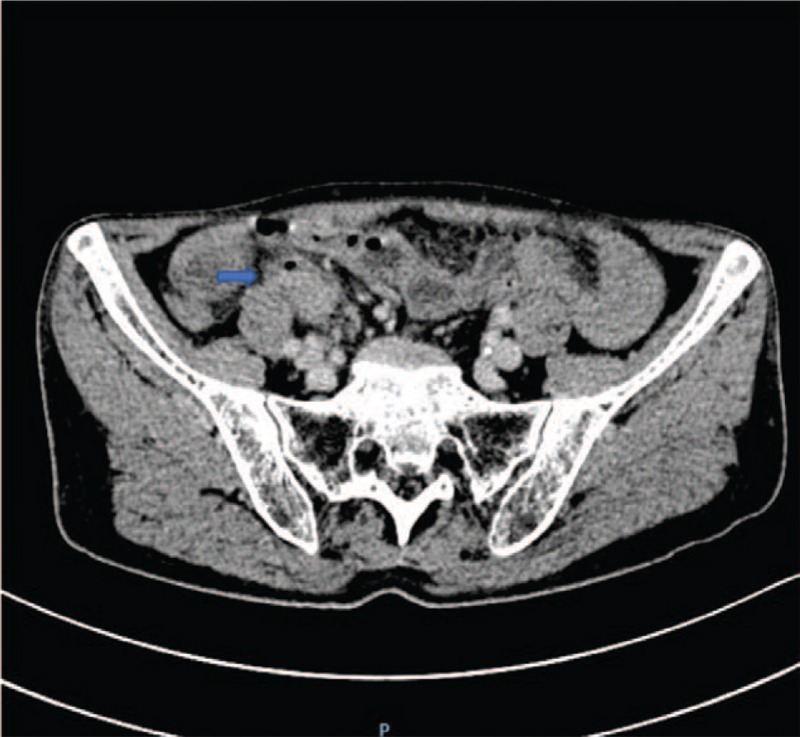
Abdominal computed tomography showed thickened bowel with enhancement (arrow indicates the abnormal bowel).

**Figure 3:**
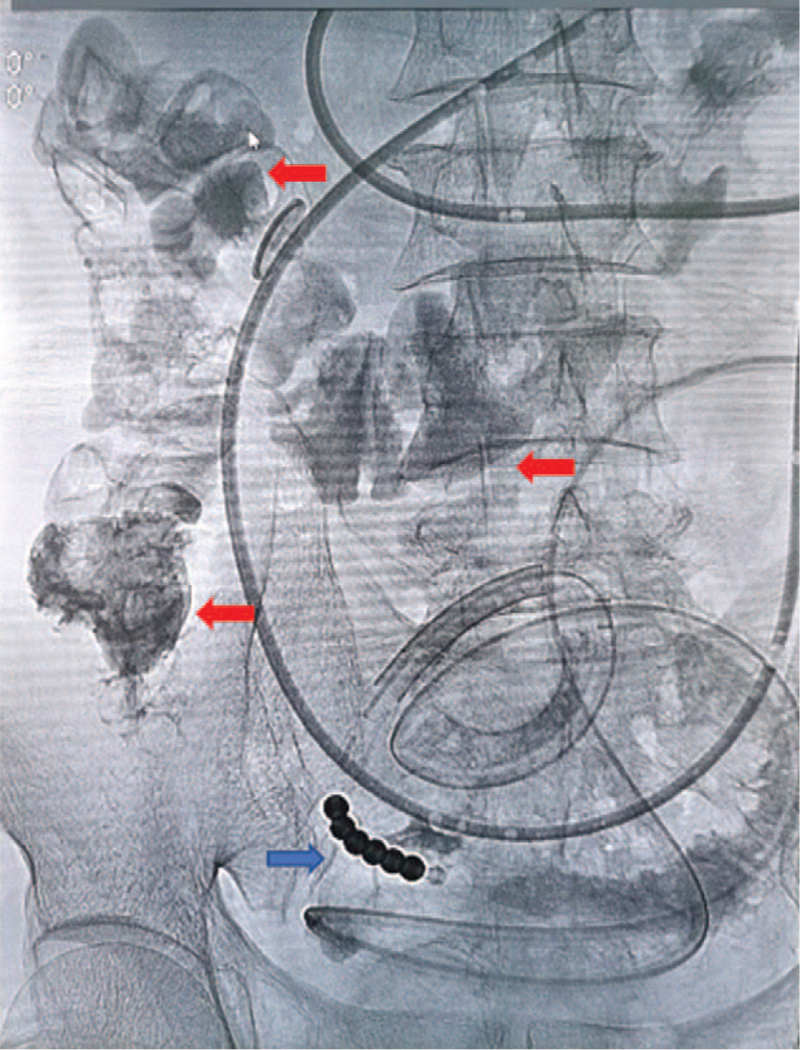
Abdominal X-ray showed the position of the ileus tube (blue arrow indicates the end of the ileus tube, red arrows indicate the visible colon).

The patient presented to our hospital on September 28, 2020, after having experienced 6 months of recurrent intestinal obstruction. Physical examination showed intestinal pattern in the lower abdomen and no obvious tenderness or rebound pain, and auscultation revealed hyperactivity of bowel sounds. Normal results were obtained on all laboratory tests, including fecal occult blood; hemoglobin (136 g/L); carcinoembryonic antigen (CEA); and carbohydrate antigens (CAs) 19-9, 72-4, and 125. Abdominal X-ray examination (Fig. 1) showed intestinal obstruction with multiple gas–liquid planes. Abdominal computed tomography (CT) revealed uneven thickening of the bowel with a narrow cavity in the right lower abdomen and local enhancement (Fig. 2). Colonoscopy showed no abnormalities. On September 29, the patient underwent placement of a transnasal ileus tube under the guidance of digital subtraction angiography. An abdominal X-ray examination was performed every 3 days to dynamically evaluate the status of the obstruction and the location of the tube. The final examination on October 5 showed that the end of the tube had reached the ileocecum, and the cecum, ascending colon, and transverse colon were visible after injection of diatrizoate meglumine (Fig. 3).

**Figure 4:**
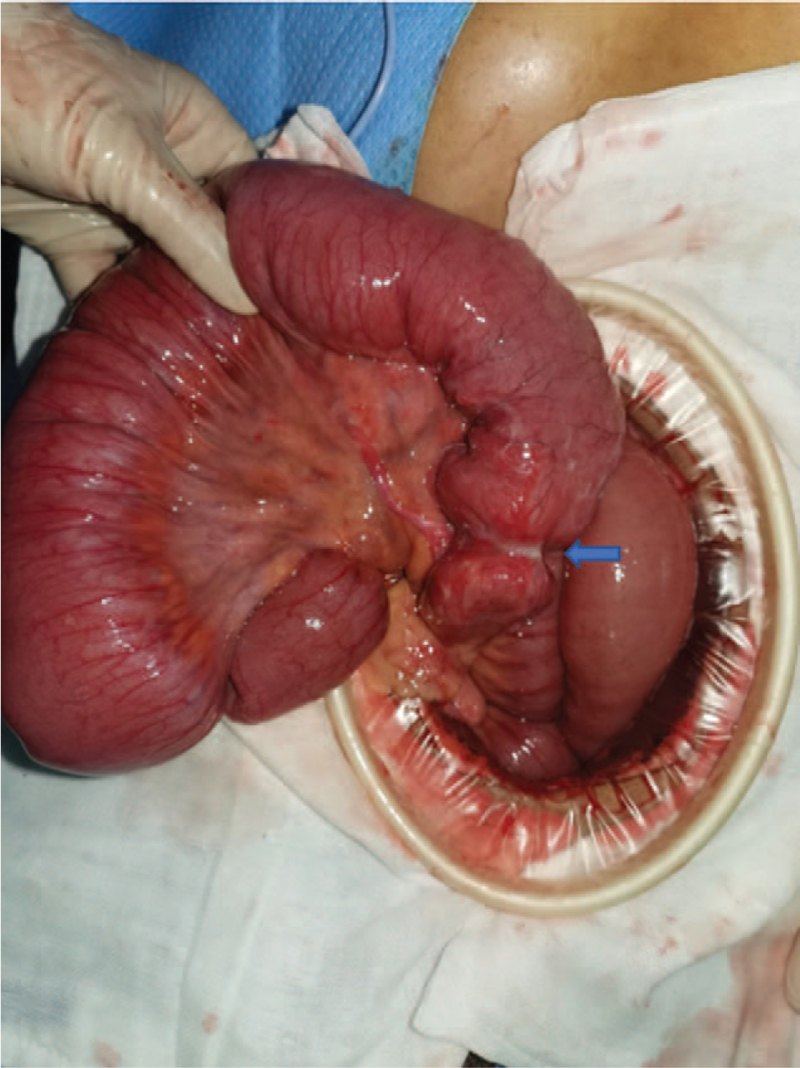
Intra-operative examination revealed small bowel adenocarcinoma in the ileum 6 cm from the ileocecal region (arrow indicates the adenocarcinoma).

The patient gradually returned to a semi-liquid diet, and exhaust and defecation became normal with no further discomfort such as abdominal pain and bloating. However, his intestinal obstruction recurred 2 weeks after discharge, and he returned to our hospital on October 31. X-ray examination revealed that the position of the ileus tube had changed. The patient underwent surgery on November 2. A tumor was found to be growing in a narrow ring cavity 6 cm from the end of the ileum to the ileocecal region, causing a complete blockage. This tumor was the direct cause of the intestinal obstruction (Fig. 4).

Multiple enlarged lymph nodes were found in the mesentery. The patient was treated by segmental resection and side-to-side anastomosis. No metastatic cancer was found in the peri-intestinal lymph nodes (0/21) or mesenteric root lymph nodes (0/3) (T3N0M0, stage IIA). The immunohistochemistry results were as follows: CK (+), CK20 (+), CDX-2 (+), MSH2 (+), MSH6 (+), PMS2 (+), MLH1 (+), Her-2 (1+), CD34 (vascular +), Smur100 (nerve +), and Ki-67 (30%–40% +). The patient recovered well after the operation. He was discharged without complications and received adjuvant chemotherapy 3 weeks after the operation (November 23, 2020). Capecitabine (1250 mg/m^2^) was taken twice daily on days 1 to 14 every 3 weeks for a total of 24 weeks without obvious adverse effects. The patient completed all postoperative adjuvant chemotherapy, and no abnormalities were found during posttreatment surveillance performed every 3 months (including a physical examination; CEA and CA19-9 measurement; and CT of the chest, abdomen, and pelvis).

## 3 Discussion and conclusions

SBA is a rare cancer that most frequently occurs in the duodenum, where it accounts for 46% to 82% of all cases, followed by the jejunum (11%–31%) and ileum (7%–21%).^[4]^ Most affected patients are asymptomatic in the early stage until complications such as bleeding, perforation, or intestinal obstruction appear, leading to a delayed diagnosis. Because of its rarity and lack of obvious symptoms, SBA is often diagnosed in its late stage, at which time 21% to 27% of patients have lymph node metastasis (stage III) and 32% to 37% of patients have distant metastasis (stage IV).^[5]^

Consumption of alcohol and certain foods, such as red meat, sugar, and starchy foods, has been reported to increase the risk of small intestinal cancer,^[6]^ whereas higher intake of coffee, fish, fruit, and vegetables is associated with a reduced risk.^[7]^ Various inherited cancer syndromes, such as familial adenomatous polyposis, Lynch syndrome, and Peutz–Jeghers syndrome, are known predisposing conditions of SBA.^[8]^ Crohn disease and celiac disease are the 2 most common diseases related to sporadic SBA, and both are associated with small intestinal inflammation.^[5]^ Patients with CRC have an increased risk of metachronous and primary colorectal tumors.^[9]^ The risk of SBA is increased following primary CRC (30% for colon cancer and 40% for rectal cancer), and the risk of CRC is 4 to 5 times higher in patients diagnosed with primary SBA.^[3]^ In the present case, the patient was finally diagnosed with metachronous SBA, and had undergone surgery and chemotherapy for rectal adenocarcinoma 30 months previously.

A study based on the National Cancer Institute's Surveillance, Epidemiology, and End Results database showed a statistically significant increase (standardized incidence ratio [SIR] = 1.15, 95% confidence interval = 1.13–1.16) in the risk of subsequent primary cancers in patients with CRC compared with the general population.^[10]^ The subsequent primary cancers included small intestinal, stomach, bladder, lung, kidney, and endometrial cancers. The incidence rate of small intestinal cancer was significantly elevated regardless of the index CRC subsite (SIR = 4.31, 95% confidence interval = 3.70–4.77).^[10]^ In a previous Surveillance, Epidemiology, and End Results analysis of the second primary non-colonic cancers after CRC, Ahmed et al^[11]^ found that the SIRs were most significant for the risk of small intestinal cancer. Research indicates that the subsequent increased risk of primary small intestinal, gastric, bladder, and lung cancer reflects the shared origin of epithelial cells derived from endoderm at these sites.^[10]^ These embryologically related tissues may have similar responses to environmental exposures and carcinogens and may be equally sensitive to abnormal epigenetic changes, which in turn form field effects, making these tissues equally prone to primary tumors.^[10]^ However, these hypotheses need to be further explored in the biological and other fields.

Multiphasic dynamic studies might improve the diagnostic accuracy of multidetector CT for small bowel neoplasms.^[5]^ Multidetector-row helical CT, with 85% to 95% overall sensitivity and 90% to 96% specificity for small bowel disease, can accurately evaluate the relationship between the tumor and adjacent blood vessels or lymph nodes. In fact, in our case, CT revealed an enhanced lesion in the bowel in the right lower abdomen; however, the radiologist and surgeon failed to clarify the specific location and diagnosis. Because SBA is rare and the patient had undergone rectal surgery, we misdiagnosed the lesion as an intestinal adhesion and began conservative treatment. The initial treatment effect was obvious, and the patient resumed his normal dietary intake. However, the obstruction later recurred because of the tumor's substantial size. The value of tumor markers in the diagnosis and follow-up of SBA is uncertain because not all patients with SBA have an elevated serum concentration of CEA or CA19-9. In the present case, the expression of these markers in the patient's serum did not change significantly. Double-balloon enteroscopy and capsule endoscopy are novel techniques for earlier diagnosis and consequent improvement of the generally poor prognosis of SBA.^[5]^ Because of the limitation of the visual range of traditional gastroenteroscopy, only tumors in the proximal duodenum and the very distal ileum can be approached by conventional endoscopy. If the colonoscope had reached the terminal ileum in our patient, an earlier and more precise diagnosis may have been achieved.

Surgical resection is still the main treatment option for patients with SBA,^[12]^ and operative techniques are selected according to the location of the tumors, the extent of tumor invasion, and the relationship of the tumor with the surrounding organs.^[13]^ For tumors of the jejunum or ileum, segmentectomy is the preferred method of resection. According to the tumor, node, metastasis (TNM) staging system,^[14]^ our patient was determined to have stage IIA (T3N0M0) cancer without high-risk features. Chemotherapy for SBA mainly refers to fluorouracil-based chemotherapy for CRC, but the efficacy is still unclear.^[5,15]^ The National Comprehensive Cancer Network Guideline (Version 1.2020) suggests either observation or 6 months of adjuvant treatment with 5-fluorouracil/leucovorin or capecitabine for T3, N0, M0 (stage IIA) tumors that are microsatellite stable or proficient mismatch repair cancers and have no high-risk features.^[16]^ Our patient received 8 cycles of adjuvant chemotherapy with capecitabine for 6 months. For SBA, an approach similar to CRC surveillance is adopted, including a history and physical examination; CEA and CA19-9 measurement; and CT of the chest, abdomen, and pelvis.^[16]^ Our patient completed all postoperative adjuvant chemotherapy, and no abnormalities were found in the posttreatment surveillance performed every 3 months.

The 5-year overall survival rate is approximately 50% to 60% for stage I SBA, 40% to 55% for stage II, 10% to 40% for stage III, and 3% to 5% for stage IV, and the prognosis of SBA appears to be intermediate between that of colon and gastric cancers.^[5]^ In addition to stage, several factors are associated with a poor prognosis, including lymph node involvement, poor histopathologic differentiation, old age, a duodenal primary tumor, positive margins, male sex, impaired performance status, black ethnicity, symptoms at the time of diagnosis, low serum albumin, high CEA or CA19-9, and high lactate dehydrogenase.^[5,16]^ Compared with CRC, SBA is more often diagnosed at advanced stages, increasing the difficulty of diagnosis and revealing the lack of screening programs, even for high-risk individuals.^[16]^ Therefore, in the clinical diagnosis of patients with any non-specific abdominal pain or unexplained anemia, small bowel tumors should be suspected. When fecal occult blood is positive, small intestinal lesions should be suspected even if no abnormality has been found by gastroenteroscopy, especially for patients with small bowel or colorectal adenocarcinoma. Close attention is needed to improve the early diagnosis rate of SBA and improve patients’ prognosis.

## Author contributions

YNZ and RXX were responsible for the coordination of the project and contributed to the study design.

YNZ, JQL, and YSZ collected and analyzed the data and edited the manuscript.

YNZ, JQL, RGW, and STL performed the operation.

YNZ, JQL, and YSZ performed the clinical work-up and therapy.

JQL and YSZ followed up the patient.

RXX performed the histological examination.

YNZ and RXX critically revised the manuscript for important intellectual content and gave final approval of the version to be published.

All authors have read and approved the final manuscript.

**Conceptualization:** Ruixue Xiao.

**Data curation:** Jianqi Li, Yongshun Zhou.

**Formal analysis:** Jianqi Li, Yongshun Zhou.

**Funding acquisition:** Ya’nan Zhen.

**Investigation:** Jianqi Li, Ruogu Wang, Shoutang Lu.

**Methodology:** Jianqi Li, Ruogu Wang, Shoutang Lu.

**Project administration:** Ya’nan Zhen.

**Writing – original draft:** Ya’nan Zhen.

**Writing – review & editing:** Ruixue Xiao.
